# Satisfactory outcomes of patellar tendon reconstruction using achilles’ tendon allograft with bone block after infected total knee arthroplasty

**DOI:** 10.1186/s40634-022-00565-w

**Published:** 2023-02-08

**Authors:** Theofylaktos Kyriakidis, Charalampos Pitsilos, Jacques Hernigou, René Verdonk, Michael Hantes

**Affiliations:** 1 Department of Orthopaedic Surgery and Traumatology, Erasme University Hospital, Université Libre de Bruxelles, Route de Lennik 808, 1070 Brussels, Belgium; 2 2nd Department of Orthopaedic Surgery and Traumatology, Aristotle University of Thessaloniki, “G. Gennimatas” General Hospital, Ethnikis Aminis 41, 54635 Thessaloniki, Hellas; 3 Department of Orthopaedics and Traumatology, Centre Hospitalier EpiCURA, Sites Hornu/Baudour, Hainaut, Belgium; 4 Department of Orthopedic Surgery, Faculty of Medicine, University of Thessaly, 41500 Larissa, Greece

## Abstract

**Purpose:**

Extensor mechanism disruption is an uncommon and devastating complication after total knee arthroplasty. It negatively affects patients’ quality of life and leads to significant functional limitations and the inability to stand and walk. The purpose of the present study was to evaluate the outcomes of the extensor mechanism reconstruction using Achilles’ tendon allograft after infected total knee arthroplasty. It was hypothesized that it is a safe procedure and that patients will present good clinical and functional results.

**Methods:**

Ten patients treated for infra-patellar extensor mechanism disruption after infected total knee arthroplasty were prospectively followed for two years. The mean age of patients was 70.8 (range 55–85), with a median BMI of 28.72 ± 2.2 kg/m^2^. All patients underwent reconstruction using a fresh frozen Achilles tendon allograft. Preoperative and postoperative evaluation included knee-related clinical and functional assessment based on objective and subjective scores, including the knee flexion, the extension lag, the Knee Society Score (KSS) clinical and functional, and the visual analog scale (VAS) for pain at 12 and 24 months. Radiological evaluation was also performed using the Caton-Deschamps index. Reported complications were also recorded.

**Results:**

Clinical and functional outcomes recorded significant improvements (*p* < 0.05) at the final follow-up compared with baseline as follows: Knee flexion was improved from 66° ± 4.8 to 99.7° ± 3.9, and the extension lag was decreased from 28.3° ± 4.4 to 9° ± 2.7. The mean KSS clinical and functional were also improved from 22.6 ± 7.9 to 73.4 ± 3.9 and from 10 ± 13.8 to 55 ± 13.8, respectively. The VAS for pain was decreased from 8.1 ± 1.2 to 1.9 ± 1.2. The Caton-Deschamps index demonstrated a tendency to patella Alta. Two treatment failures were recorded, one patellar dislocation and one re-rupture revised to arthrodesis.

**Conclusions:**

Extensor mechanism reconstruction using Achilles’ tendon allograft after infected total knee arthroplasty is an efficient and safe procedure for infra-patellar disruption. The present study’s findings demonstrate that most of the patients (80%) presented significant clinical and functional improvement at two-year follow-up.

## Introduction

Patients presenting extensor mechanism (ΕΜ) disruption due to infection after total knee arthroplasty (TKA) require a highly challenging injury treatment [2]. The majority of them have numerous previous surgical procedures which negatively affect the soft tissue quality and lead to significant functional limitations and inability to stand and walk [16].

Indeed, extensor mechanism disruption is a severe and challenging complication following total knee arthroplasty leading to chronic pain, limited range of motion, extension lag, and gait abnormalities [13] and most often occurs as a patellar tendon rupture [9]. The reported incidence is between 0.1% and 2.5%, and the commonly suggested predisposing factors include multiple prior operations, stiffness, patella baja, obesity, systemic pathologies or medications, and infections [18].

Multiple treatment modalities are available to cope with these significant complications, including two-stage revision arthroplasty combined with extensor mechanism reconstruction, knee arthrodesis, or amputation above the knee [15, 17]. However, there is still no clear consensus on treating these patients, and limited data exist in the literature to evaluate their functional outcomes [24].

Either pre-or post-arthroplasty infection is another devastating complication that harms patients' quality of life and autonomy and leads to soft tissue degeneration and, in time, extensor mechanism disruption necessitating revision surgery [22]. The admixture of these complications is rare, making treatment even more challenging for both the patient and the surgeon.

Surgical reconstruction of the extensor mechanism, remains challenging and is considered a salvage procedure to avoid the functional limitation of arthrodesis or amputation and maintain a greater level of knee function. This technique mainly includes synthetic material or allografts, presenting acceptable results [11]. Most often utilized allografts are either the whole extensor mechanism comprising quadriceps tendon, patella, patellar tendon, tibial tubercule block, or the Achilles’ tendon with calcaneum bone block [5, 23].

However, to our best knowledge, no clinical study examined reconstruction with Achilles’ tendon allograft in the case of previously infected total knee arthroplasty. The purpose of the present study was to evaluate the outcomes of extensor mechanism reconstruction for infrapatellar disruption using Achilles’ tendon allograft after infected total knee arthroplasty. It was hypothesized that this procedure could provide good clinical and functional outcomes.

### Materials and methods

The study included ten patients treated for infra-patellar extensor mechanism rupture using Achilles’ tendon allograft after infected total knee arthroplasty between 2015 and 2019. The diagnosis of infected total knee arthroplasty was made according to the International Consensus on Periprosthetic Joint Infection criteria [19] or its later modification [20].

All patients reported an inability to walk and to do straight leg raise. The extensor mechanism rupture was diagnosed either clinically and confirmed echographically or intraoperatively during the prior surgical procedures. It is noted that the insufficiency of the extensor mechanism was identified after the initial diagnosis of infection as part of the infection process. The patients had multiple previous surgeries as shown in Table 2, and the salvage procedure consisting in the revision of the total knee arthroplasty combined with the extensor mechanism allograft reconstruction was performed at least three months after infection eradication.

After the diagnosis of the infection, the first step of the treatment was the removal of the initial components and cement, extensive debridement of the joint cavity, and implantation of an antibiotic-impregnated (Gentamycin) articulating spacer.

The complete inclusion and exclusion criteria are listed in Table 1. Analytical demographic characteristics of the study population are provided in Table 2.

**Table 1:** Inclusion and exclusion criteria of eligibility

*Inclusion Criteria*
* 1. Infra patellar extensor mechanism disruption after infected TKA*
* 2. Male and female gender with primary TKA implants*
* 3. Body Mass Index (BMI)* ≤ *35 kg/m*^*2*^*)*
* 4. Inability to walk and straight leg raise*
* 5. Minimum 3 months after infection eradication*
*Exclusion criteria*
* 1. Patients with revision implants*
* 2. Active septic arthritis*
* 3. Previous extensor mechanism reconstruction surgery*
* 4. Patients unable to follow the rehabilitation protocol*
* 5. Good tissue quality permitting autograft reconstruction*

**Table 2:** Patient’s demographic characteristics

Participants	10
Gender	Female, *n* = 6 Male, *n* = 4
Age	70.8 years (range 55–85)
BMI	27.7 ± 2.2 kg/m^2^
Complications	1 Patellar dislocation, 1 Arthrodesis
Previous procedures	5–9 (meniscectomy, TKA, RTKA, spacer implantation, arthroscopic or open debridement)
ASA Classification	II, *n* = 3 III, *n* = 7
Isolated Microorganisms	Staphylococcus aureus × 4, Staphylococcus Epidermidis × 3, Serratia marcescens, Pseudomonas, Enterobacter
Time to diagnosis of infection	38 months (range 6–84)
Time to diagnosis of tendon rupture	42 months (range 12–86)
Time to reconstruction	47 months (range 18–89)

*TKA* Total knee arthroplasty, *RTKA* Revision total knee arthroplasty

### Surgical technique

**Fig. 1:**
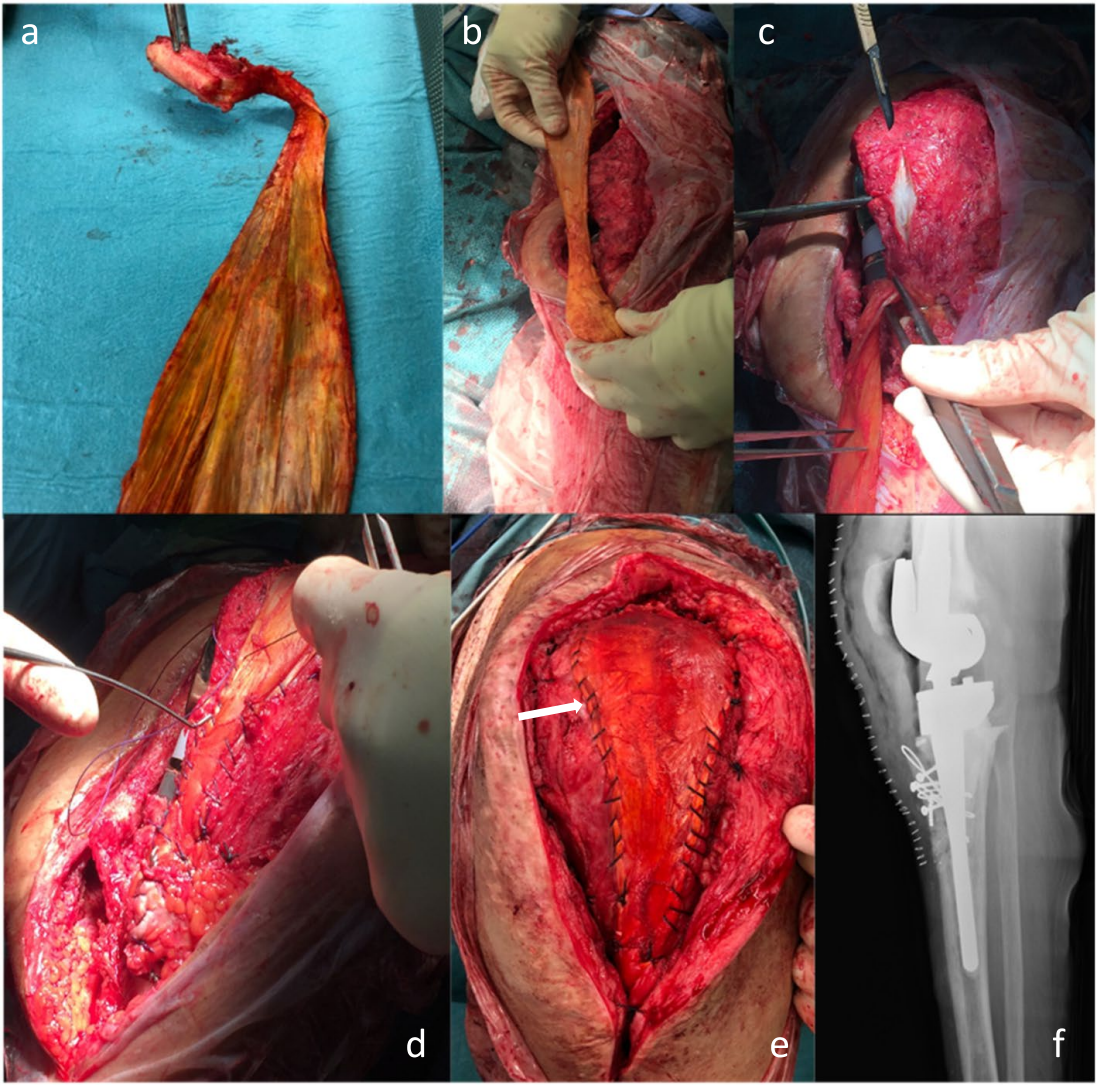
Different steps of extensor mechanism reconstruction. a: Achilles’ tendon allograft, b: graft preparation, c: patellar tendon split, d: the Achilles’ tendon was sutured with a number 2 nonabsorbable sutures in full extension, e: final placement of the graft, f: Radiographic evaluation

Classical revision total knee arthroplasty principles were followed for proper prosthesis implantation using rotating modular hinge design implants. Extensor mechanism reconstruction was performed at the same surgical time as the revision implantation. A 24–26 cm long fresh-frozen Achilles’ tendon allograft with bone attachment to the calcaneum was used (Fig. 1a, b). One of the previous incisions was used to expose the whole extensor apparatus extensively. A medial parapatellar arthrotomy was performed. After the final placement of the components, a rectangular anterior tibial bone “window” of approximately 3 cm long by 2 cm wide and 1,5 cm deep was performed just inferior to the patellar tendon insertion at the anterior tibial tubercle. The calcaneal bone block was prepared to fit in the “window.” It was softly impacted into the tibial defect, and then it was fixed with 4.0 mm screws and metallic cerclages. The screw direction was angled to avoid the tibial stem. The patella was centered in the trochlea and verified in full extension and 30 degrees of flexion to achieve the optimal height. A split to the patellar tendon was performed (Fig. 1c), and the Achilles’ tendon was sutured with a number 2 nonabsorbable sutures in full extension to the underlying extensor mechanism (Fig. 1 d, e). Subcutaneous tissue and skin closure were completed, and a compressive dressing was applied. Radiological evaluation was performed (Fig. 1.f).

### Postoperative protocol

After surgery, all patients were placed in an extension brace for four weeks and allowed weight bearing as tolerated. No active straight leg raises, or quad sets were permitted during this period. A functional brace was placed from the 5th week, allowing an active flexion up to 30° initially and then gradually increased each week for 15°. At two months, post-operative patients were allowed a range of motion up to 90° with full weight-bearing. Straight leg raises exercises were also allowed. The brace was removed three months after surgery, and the range of motion was unrestricted.

### Evaluation of outcomes

All patients were clinically evaluated at 12 and 24 months. Primary outcomes were the knee flexion and the extension lag measured with a manual goniometer with the patient in the supine position from the lateral side of the knee. Secondary outcomes included the Knee Society Score (KSS) clinical and functional, the visual analog scale (VAS) for pain. Clinical failure was defined as an extension lag higher than 20° or cases needing revision surgery. Complications were recorded as well. Standard radiological evaluation including anteroposterior, lateral and patellar views was performed in all the follow-up intervals. The patellar height was also calculated using the Caton-Deschamps index. All patients agreed to follow the same rehabilitation protocol.

### Statistical analysis

Statistical analysis was performed by an independent statistician using IBM SPSS Statistics (IBM Corp. Released 2020. IBM SPSS Statistics for Windows, Version 27.0. Armonk, NY: IBM Corp). One-way repeated measures ANOVA was applied to analyze outcomes between pre-operative and post-operative findings after checking normality assumptions using the Shapiro–Wilk test. If the condition of sphericity had not been met, the *p*-values were adjusted according to Greenhouse–Geisser correction. As the within-subjects ANOVA showed a significant difference, pairwise comparisons were used, applying Bonferroni corrections. The significance level was set at 5%.

## Results

**Fig. 2:**
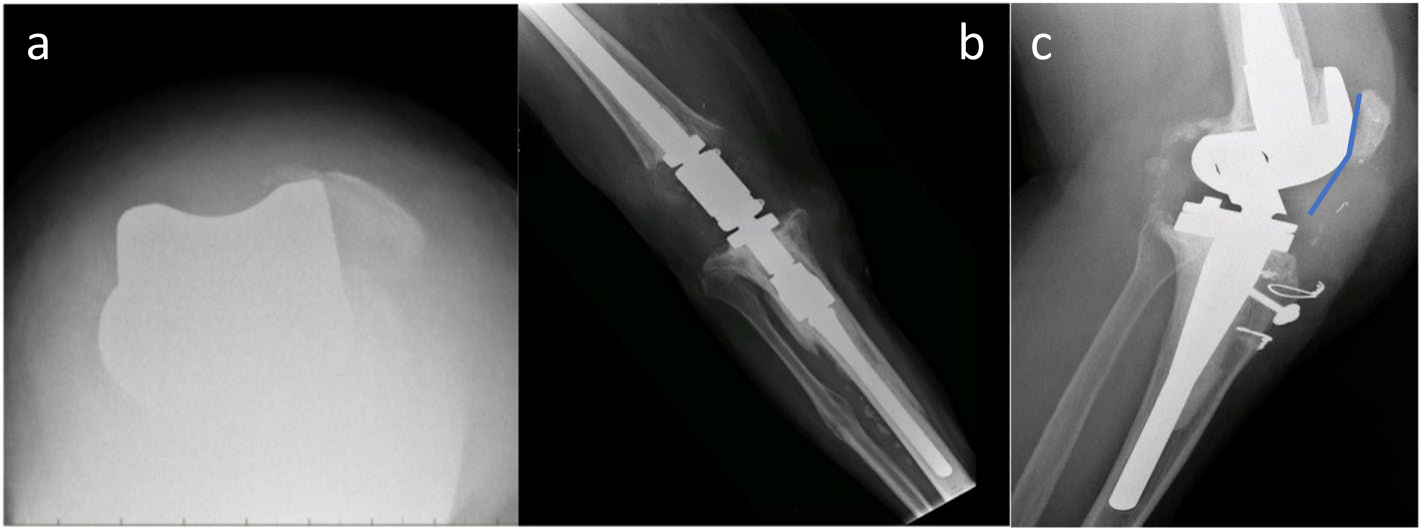
a. X-ray of patellar dislocation b. X-ray of knee arthrodesis c. Caton-Deschamps index

All patients who met the eligibility criteria were included in this study and prospectively followed for at least 24 months. The mean follow-up was of 34 months (range 24–53 months). Complete data were recorded for all patients. The patients had many previous surgical procedures varied from 5 to 9. These procedures comprise partial meniscectomy, primary total arthroplasty, revision total knee arthroplasty, arthroscopic or open debridement, and spacer implantation. Statistically, significant improvement was found in all clinical and functional assessment tools (*p* < 0.05). More precisely, the knee flexion was increased, reaching a final value of approximately 100°, while the extension lag was decreased to less than 10°. KSS clinical and functional scores were 73.4 and 50, respectively, and VAS for pain was about 2 at the last follow-up. Detailed outcomes are provided in Table 3. Radiological evaluation revealed no sign of implant failure or periprosthetic fracture. Two treatment failures were recorded and thus, excluded from further analysis. One patient had a postoperative patellar dislocation (Fig. 2a) which negatively affected extensor mechanism function, and another one had a re-rupture of the extensor mechanism requiring a revision to arthrodesis (Fig. 2b). The remaining patients were satisfied as they could actively raise their legs and climb stairs without crutches. The mean Caton-Deschamps index was 1.5 and demonstrated a tendance to a patella alta (Fig. 2c).

**Table 3:** Summary of Outcomes

Outcomes	Preoperative	12 Months FU	24 Months FU	*P* values		
				Preop vs 12 m	Preop vs 24 m	12 m vs 24 m
Flexion	66^o^ ± 4.8	99.8^o^ ± 3.8	99.7^o^ ± 3.9	*p* < 0.05	*p* < 0.05	NS
Extension lag	28.3^o^ ± 4.4	9.1^o^ ± 2.4	9^o^ ± 2.7	*p* < 0.05	*p* < 0.05	NS
KSS clinical	22.6 ± 7.9	73.8 ± 5.9	73.4 ± 3.9	*p* < 0.05	*p* < 0.05	NS
KSS function	10 ± 13.8	50 ± 8.9	50 ± 8.9	*p* < 0.05	*p* < 0.05	NS
VAS pain	8.1 ± 1.2	1.9 ± 1.3	1.9 ± 1.2	*p* < 0.05	*p* < 0.05	NS

All outcome values are described as mean ± standard deviation

*ROM* Range of motion, *KSS* Knee society Score, *VAS* Visual Analogue Scale, *NS* Not significant, *vs* versus

## Discussion

The most important finding of the present study was that extensor mechanism reconstruction following infected total knee arthroplasty with Achilles’ tendon allograft was shown to be an efficient and safe procedure in the majority of patients with good clinical results within two-year of follow-up. Nevertheless, 20% of the patient presented a treatment failure.

The overall outcome was satisfactory, with a significant improvement in knee flexion, extension lag, KSS clinical and functional scores and VAS for pain. Radiological evaluation revealed a tendance to patella alta according to the Caton-Deschamps index.

Previous studies reported surgical reconstruction outcomes using synthetic material or allografts after total knee arthroplasty [1, 6, 13, 14]. Achilles’ tendon allograft with calcaneal bone block has been widely used for disrupted patellar tendon reconstruction after aseptic TKA and presented the most successful results [10, 13, 21, 25].

For instance, Crossett et al. [10] studied nine patients with a mean follow-up of 28 months. They found that the average postoperative extensor lag had decreased from 44° to 3°, with six patients demonstrating no extensor lag, and the average range of motion had increased from 88^0^ to 107°. Moreover, all patients reported improved ability to walk and climb stairs. They concluded that it could be a faithful reconstruction of a ruptured patellar tendon and may be particularly suited for patients in whom multiple prior operations compromised the extensor mechanism.

Additionally, Wise et al. [25] reported that Achilles’ tendon allograft is a reliable and durable treatment for patients presenting extensor mechanism disruption following TKA. They retrospectively evaluated seventeen cases of extensor mechanism failure following total knee arthroplasty in sixteen patients who underwent reconstruction using Achilles’ tendon allograft assessed at an average of 45.7 months. The ten cases involved patellar tendon disruption, and the study demonstrated an average postoperative extensor lag and flexion of 9.6° and 105.1°, respectively.

To date, limited data exist in allograft reconstruction after infected total knee arthroplasty. Indeed, no recent studies focus only on this demanding population. Even so, some authors include limited cases in previous case series [3, 12].

In fact, Lamberti et al. [12] compared mid-term results of three different reconstructive techniques for chronic patellar rupture. Patients underwent reconstruction either with extensor mechanism or Achilles’ tendon allograft or quadriceps tendon autograft with semitendinosus tendon augmentation. They included seven patients in each group, and they reported statistically significant changes in all groups for the mean knee society score (KSS) and the mean extension lag. However, patients treated with Achilles’ tendon allograft presented better improvement from 34.9 ± 21.3 to 87.7 ± 14.3 and from 54° ± 17.2° to 2° ± 1.5°, respectively. Thus, the authors consider this technique the gold standard for repairing a chronic patellar tendon lesion after total knee arthroplasty. Nevertheless, it should be noted that only two out of the seven patients in this group presented a previous periprosthetic infection.

Ares et al. [3] reported an observational study of five patients diagnosed with patellar tendon rupture after total knee replacement and surgically treated with Achilles’ tendon allograft reconstruction. However, only one patient had a prior infection. All patients presented a mean extensor lag of 1^ο^ and flexion of 102°, and they were able to walk without crutches at a mean of 25 months postoperatively. The only complication recorded was one patellofemoral pain syndrome.

The present study evaluated ten patients treated with Achilles’ tendon allograft reconstruction for infrapatellar rupture after septic TKA. An improvement was observed both in clinical and functional outcomes. Moreover, KSS clinical and functional scores were improved, reaching at the final follow-up 73.4 and 50 points, respectively. Finally, the VAS score was decreased from 8 to 2. These outcomes indicate a notable change in the patient’s quality of life.

These findings are in accordance with previously reported data presenting the Achilles tendon allograft as an effective treatment for reconstructing ruptured patellar tendon [8, 10, 25]. Barrack et al. [4] reported a summary average of clinical and functional Knee Society Score, which improved from 32 to 128 points, representing a highly significant improvement. Crosset et al. [10], in their retrospective study, included nine patients treated for acute or chronic patellar tendon rupture after total or revision knee arthroplasty. They observed an improvement in KSS clinical and functional scores from 26 to 81 and from 14 to 53 points, respectively. This improvement is slightly superior to that in the present study. However, it should be noted that their study included only two cases with a prior deep infection, which partly could explain the results.

Two complications were recorded in the current study. It has been well documented that the initial allografts’ tensioning is crucial to avoiding persistent extension lag and clinical failure [7]. Therefore, all the allografts were sutured under high tension in full extension. However, a patellar dislocation due to sutures loosening was observed. Thus, very rigid fixation of the graft could be complicated with a suture loosening in patients presenting poor soft tissue quality due to numerous previous procedures. The second complication was a re-rupture of the extensor mechanism following low energy trauma and treated with knee arthrodesis.

There are also specific limitations that have to be considered in the current study. This study is a case series with a low number of participants and without a comparative group of other possible grafts and thus involved selection bias. Nevertheless, due to septic total knee arthroplasty, extensor mechanism disruption is a rare complication that makes difficult the inclusion of patients in this type of study. Hence, future studies with more patients and a longer follow-up are needed to validate these data. Another limitation is the absence of cost-effective and psychological aspects analysis. Moreover, the limited follow-up period after this procedure is recognized as another potential limitation.

## Conclusion

Extensor mechanism reconstruction using Achilles' tendon allograft after infected total knee arthroplasty is an efficient and safe procedure for infrapatellar disruption. The present study's findings demonstrate that the majority of the patients presented significant clinical and functional improvement within two-year of follow-up. However, we recorded 20% of treatment failure.

## Data Availability

The datasets used and/or analysed during the current study are available from the corresponding author on reasonable request.
